# Elevated Serum Regulator of Calcineurin 2 is Associated With an Increased Risk of Non-Alcoholic Fatty Liver Disease

**DOI:** 10.3389/fphar.2022.840764

**Published:** 2022-03-16

**Authors:** Xia Fang, Hongya Wang, Xiaozhen Tan, Ting Ye, Yong Xu, Jiahao Fan

**Affiliations:** ^1^ Department of Endocrinology and Metabolism, The Affiliated Hospital of Southwest Medical University, Luzhou, China; ^2^ Metabolic Vascular Disease Key Laboratory of Sichuan Province, Luzhou, China; ^3^ Sichuan Clinical Research Center for Nephropathy, Luzhou, China; ^4^ Cardiovascular and Metabolic Diseases Key Laboratory of Luzhou, Luzhou, China; ^5^ Department of Laboratory Medicine, The Affiliated Hospital of Southwest Medical University, Luzhou, China; ^6^ Department of Gastroenterology, The Affiliated Hospital of Southwest Medical University, Luzhou, China

**Keywords:** non-alcoholic fatty liver disease, regulator of calcineurin 2, cross-sectional, biomarker, Chinese

## Abstract

**Background:** The promoting effect of the regulator of calcineurin 2 (RCAN2) in hepatic steatosis has been observed in animal studies. However, the association of RCAN2 with non-alcoholic fatty liver disease (NAFLD) in humans remains unclear. This study aimed to evaluate the expression of RCAN2 in the liver of mice with hepatic steatosis and in the serum of NAFLD patients and to explore the relationship between serum RCAN2 levels and NAFLD.

**Methods:** The mRNA and protein expression of RCAN2 were detected by quantitative real-time PCR (qRT-PCR) and Western blot. NAFLD was diagnosed by abdominal ultrasonography. Circulating RCAN2 levels were measured by ELISA kits. The relationship between serum RCAN2 levels and NAFLD was assessed.

**Results:** qRT-PCR and Western blot analysis showed that compared with the corresponding controls, the mRNA and protein expression of RCAN2 were significantly increased in the liver tissues of db/db and mice on a high-fat diet. Serum RCAN2 levels were markedly elevated in NAFLD patients compared with non-NAFLD subjects. Binary logistic regression analysis showed that serum RCAN2 levels were significantly associated with NAFLD. Receiver operation characteristic (ROC) curve analysis showed that serum RCAN2 might act as a predictive biomarker for NAFLD [area under the curve (AUC) = 0.663, 95% CI = 0.623–0.702], and the serum RCAN2/(AST/ALT) ratio displayed improved predictive accuracy (AUC = 0.816, 95% CI = 0.785–0.846).

**Conclusion:** Elevated serum RCAN2 levels were associated with an increased risk of NAFLD. Serum RCAN2, especially the serum RCAN2/(AST/ALT) ratio, might be a candidate diagnostic marker for NAFLD.

## Introduction

Non-alcoholic fatty liver disease (NAFLD) is defined as the presence of steatosis in more than 5% of the hepatocytes without other clear causes of liver fat accumulation, such as viruses, alcohol, drugs, or genetic factors (Chalasani et al., 2012). NAFLD comprises simple hepatic steatosis (fatty liver) alone and nonalcoholic steatohepatitis (NASH), a more serious process with inflammation, hepatocyte damage, or fibrosis that can eventually progress to liver cirrhosis and hepatocellular carcinoma (HCC) (Chalasani et al., 2018; Parthasarathy et al., 2020). In recent decades, NAFLD has become a major public health concern. The number of NAFLD patients is dramatically increasing worldwide, and the global prevalence of NAFLD has been estimated to be about 25% (Younossi et al., 2016; Negro, 2020). NAFLD has emerged as one of the predominant causes of chronic liver diseases worldwide. Its pathogenesis is complicated, including increased *de novo* synthesis of liver fatty acids, hepatocellular injury, liver insulin resistance, intestinal dysbiosis, and fibrogenesis (Friedman et al., 2018). However, these mechanisms have not been well elucidated, and there are currently no effective drugs for the treatment of NAFLD or approved biomarkers to assess disease progression. Although some studies have evaluated potential biomarkers, they have been rarely verified and seldom used in clinical practice. Therefore, there is still a great need to identify and validate other effective biomarkers to predict NAFLD.

The regulator of calcineurin 2 (RCAN2) [also termed thyroid hormone-responsive protein ZAKI-4, down syndrome candidate region 1-like 1 (DSCR1L1), or myocyte-enriched calcineurin-interacting protein 2 (MCIP2)] (Davies et al., 2007) was initially identified as a thyroid hormone (triiodothyronine, T3)-responsive gene in human skin fibroblasts (Miyazaki et al., 1996) and then reported to inhibit calcineurin-dependent transcriptional responses by binding to the catalytic subunit A of calcineurin (Fuentes et al., 2000; Kingsbury and Cunningham, 2000). ZAKI-4 has three transcripts, α (also called RCAN2-3), β1, and β2, in which β1 and β2 encode an identical protein product β (also called RCAN2-1) (Cao et al., 2002). Mouse RCAN2 isoforms are highly homologous with human transcripts. The RCAN2 mouse gene, located on chromosome 17, corresponds exactly to the gene containing the corresponding human homolog on chromosome 6 (Strippoli et al., 2000). The tissue distribution of RCAN2 isoforms in mice is highly consistent with that in humans. RCAN2-3 is expressed only in the brain, while RCAN2-1 is ubiquitously expressed in the brain, heart, muscle, kidney, and liver (Cao et al., 2002; Mizuno et al., 2004). RCAN2 is involved in many pathophysiological processes. In striated muscles, RCAN2 modulates hypertrophic growth and selective programs of gene expression (Rothermel et al., 2000). RCAN2 plays a vital role in the brain development and function by regulating the function of calcineurin (Siddiq et al., 2001). RCAN2 is expressed in endothelial cells and restricts angiogenesis by inhibiting calcineurin activity (Gollogly et al., 2007). RCAN2 is also involved in the development, proliferation, or pro-tumorigenic network of cancers (Niitsu et al., 2016; Hattori et al., 2019; Mammarella et al., 2021). Interestingly, the knockout of RCAN2 in the whole body can significantly reduce the liver weight of high-fat diet-induced obese mice and did not exhibit liver steatosis (Sun et al., 2011; Zhao et al., 2016). This suggests that RCAN2 plays an important role in NAFLD in mice; however, there is still no evidence that RCAN2 is involved in the development of human NAFLD or that it may be a diagnostic marker for NAFLD.

In the present study, we aimed to conduct the first study to evaluate serum RCAN2 levels in NAFLD patients and non-NAFLD subjects, and the association of serum RCAN2 with NAFLD risk.

## Materials and Methods

### Animal Models and Diet

Six-week-old male C57BL/6 mice, db/db mice, and heterozygous control db/m mice were purchased from SPF Biotechnology Co., Ltd. (Beijing, China). All mice were housed in the IVC system with a 12-h light/dark cycles and ad libitum access to food and water. After a 7-d acclimation, 7-week-old male C57BL/6 mice were fed a high-fat diet (HFD) for 16 weeks (containing 60.0% fat, 20.6% carbohydrate, and 19.4% protein; TP23300) to induce fatty liver or low-fat diet (LFD; TP23302) as controls. HFD and LFD were purchased from Trophic Animal Feed High-Tech Co., Ltd., Nantong, China. The db/db mice served as another fatty liver model and were fed a normal diet for 6 weeks. All animals were sacrificed under anesthesia and the liver tissues were excised and frozen at −80°C for analyzing the mRNA and protein levels. All animal protocols were approved by the Experimental Animal Ethics Committee of Southwest Medical University (Permission no. 2020827).

### Liver Histological Analysis, Quantitative Real-Time PCR, and Western Blot Analyses

The liver was fixed overnight with 10% phosphate-buffered formalin acetate at 4°C and embedded in paraffin. The paraffin sections were mounted on glass slides for hematoxylin and eosin (H&E) staining. The total RNA was extracted from the liver tissues using TRIzol reagent (15596026, Thermo Fisher), and then the total RNA was reverse transcribed to cDNA using the first strand cDNA synthesis kit (FSQ-201, Toyobo). qRT-PCR of cDNA was performed using SYBR Green PCR Master Mix (208056, Qiagen) in a StepOnePlus Real-Time PCR System (qTOWER^3^ G, Germany). The relative mRNA expression levels of the target genes were normalized to GAPDH expression. The primer sequences are shown in Supplementary Table S1. Western blots were used for protein expression, as described previously (Gao et al., 2019). Primary antibodies were anti-RCAN2 (1:3,000, Thermo Fisher Scientific, PA5-112914) and anti-tubulin (1:5,000, Beyotime, China, AF5012). Western blots were visualized by a chemiluminescence system (ChemStudio_815, Germany) and quantified by using the ImageJ software (National Institutes of Health, Bethesda, MD).

### Study Participants

All participants were recruited from the physical examination center of The Affiliated Hospital of Southwest Medical University. Fatty liver can be diagnosed by abdominal ultrasonography with two of the following three abnormalities: 1) diffusely increased liver tissue near the field ultrasonic echo; 2) kidney echo weaker than liver; and 3) vascular blurring and the gradual attenuation of the far-field ultrasonic echo (Fan et al., 2011). Fatty liver can be divided into mild (ultrasonic echo attenuation does not exceed one-third of the liver), moderate (ultrasonic echo attenuation exceeds one-third and less than two-thirds of the liver), and severe (ultrasonic echo attenuation exceeds two-thirds of the liver). The subjects were excluded if they had any of the following: liver dysfunction caused by other reasons (e.g., alcohol, drugs, toxins, virus, or genetic factors) other than NAFLD, cancer, kidney dysfunction, cardiovascular or cerebrovascular disease, history of thyroid disease, HIV infection, pregnancy or breastfeeding, systemic corticosteroid treatment, anti-inflammatory therapy, and hypoglycemic, lipid-lowering, or antihypertensive treatment. All human investigations followed the ethical guidelines of the 1964 Declaration of Helsinki and were approved by the Human Research Ethics Committee of the Affiliated Hospital of Southwest Medical University (Permission no. KY2021086). Informed consent was obtained from all participants in this study.

### Anthropometric and Biochemical Measurements and Serum Sample Collection

After overnight fasting, all anthropometric parameters such as height, body weight (BW), waist circumference (WC), hip circumference (HC), systolic blood pressure (SBP), and diastolic blood pressure (DBP) were measured by professionally trained nurses. The body mass index (BMI) was calculated as BW divided by height squared. The waist-to-hip ratio (WHR) was calculated by WC and HC. Blood indexes such as fasting blood glucose (FBG), alanine aminotransferase (ALT), aspartate aminotransferase (AST), gamma-glutamyl transpeptidase (GGT), alkaline phosphatase (ALP), urea nitrogen (UREA), uric acid (UA), creatinine (CREA), total cholesterol (TC), triglycerides (TG), high-density lipoprotein cholesterol (HDL-C), low-density lipoprotein cholesterol (LDL-C), homocysteine (HCY), and free triiodothyronine (FT3) were tested in the department of laboratory medicine and nuclear medicine of the Affiliated Hospital of Southwest Medical University. The fatty liver index (FLI) was calculated using the following formulaa: FLI = 1/[1 + exp(−x)] × 100, x = 0.953 × ln (triglycerides) (mg/dl) + 0.139 × BMI (kg/m2) + 0.718 × ln (γ-glutamyl-transferase) (U/L) + 0.053 × WC (cm)–15.745 (Tan et al., 2021). After overnight fasting, the blood samples from all the participants were collected in the separation gel promoting tubes on the morning of the day of ultrasound examination. The serum was collected after centrifugation at 3,000 rpm at 4°C for 15 min. To avoid repeated freeze-thaw cycles, each sample was divided into several tubes and stored at −80°C until analysis.

### Measurements of Serum RCAN2

Serum RCAN2 levels were measured with a commercial enzyme-linked immunosorbent assay (ELISA) kit (Human ELISA kit, AE22621HU; Mouse ELISA Kit, AE22620MO, Abebio, China) according to the manufacturer’s protocol. The pilot experiments showed that RCAN2 can be detected in our 10-fold diluted human serum samples and in 100 μL mouse serum samples. Therefore, all human serum samples were diluted 10-fold before detection, while mouse serum samples were tested with 100 μL. The intra- and inter-assay variations were <8% and <12%, respectively.

### Statistical Analysis

SPSS 22.0 and GraphPad Prism 8.0 were used for all statistical analysis and graphics. Data were expressed as number (%) for categorical variables or as mean ± standard deviation (SD) for continuous variables. For continuous variables, Student’s t-test or the Mann-Whitney U-test was used for the differences between the two groups, and one-way analysis of variance (ANOVA) or the Kruskal-Wallis test was used for the differences among more than two groups. Comparisons of categorical variables were made using the Chi-square test. The correlation between serum RCAN2 and other clinical parameters was determined by partial correlation coefficients. Variables independently associated with serum RCAN2 were analyzed by multiple linear regressions. Binary logistic regression analyses were used to analyze the association between the serum RCAN2 levels and NAFLD risk. The diagnostic value of serum RCAN2 or serum RCAN2/(AST/ALT) ratio for the NAFLD was evaluated by the area under the receiver operating characteristic (ROC) curve (AUC). A *p*-value < 0.05 was considered statistically significant.

## Results

### Hepatic RCAN2 Expression Was Up-Regulated in Db/db and HFD-induced Hepatic Steatosis Mice

**FIGURE 1:**
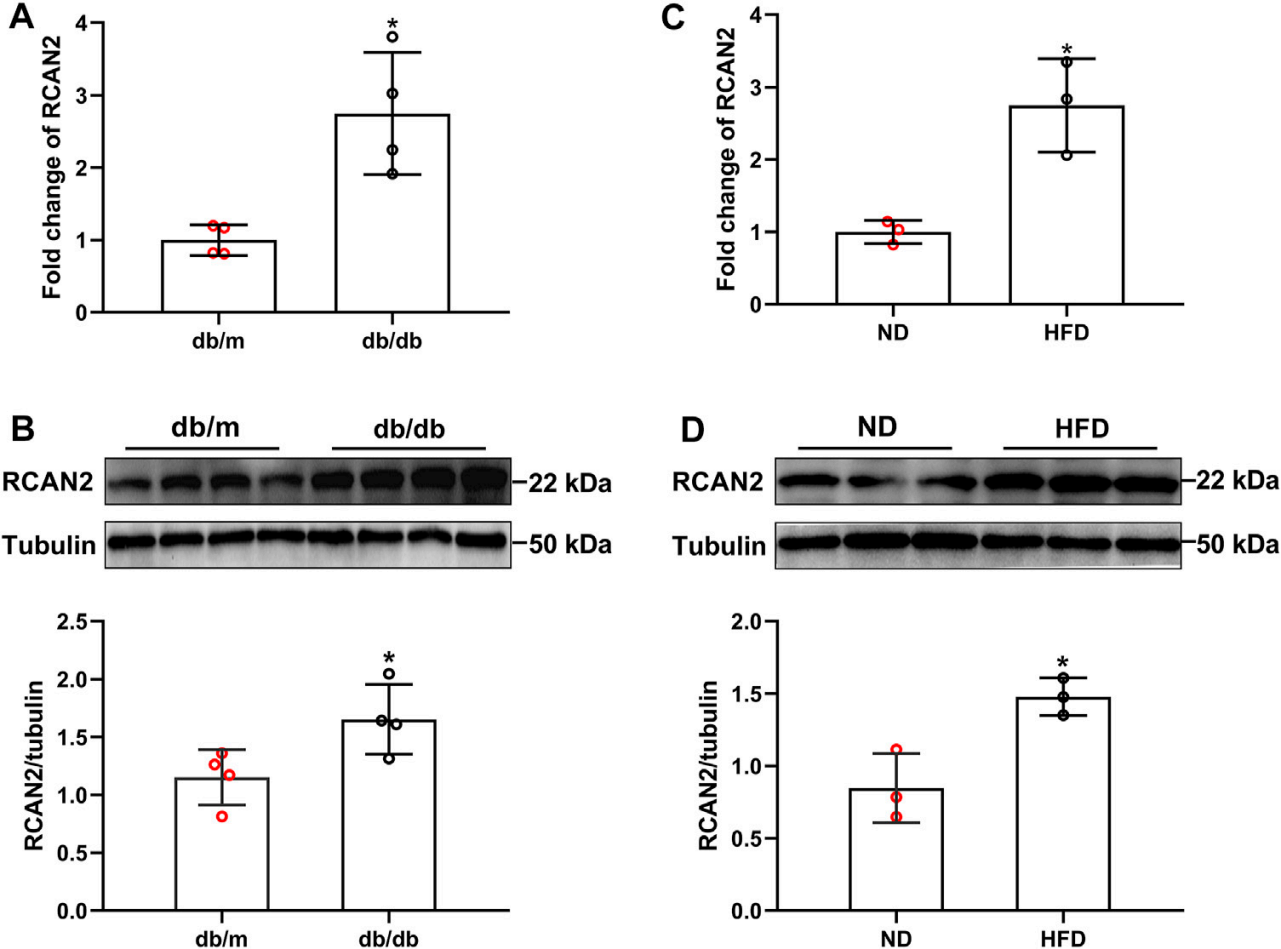
RCAN2 expression was upregulated in livers of db/db mice and HFD-induced mice. The mRNA **(A)** and protein **(B)** expression of RCAN2 in db/db mice vs db/m mice; The mRNA **(C)** and protein **(D)** expression of RCAN2 in HFD mice vs ND mice. ND, normal diet; HFD, high-fat diet. The data was presented as mean ± SD. *p < 0.05 vs ND or db/m.

As shown in the Supplementary Figures S1, S2, we successfully constructed a mouse model of hepatic steatosis. In order to explore the potential role of RCAN2 in the development of fatty liver, we first analyzed its expression in the liver tissues of mice. Our data showed that compared with db/m mice, the mRNA and protein expression levels were significantly increased in the liver tissues of db/db mice (Figures 1A,B). Elevated RCAN2 mRNA and protein expression was also observed in the liver tissues of HFD-induced fatty liver mice (Figures 1C,D). We detected the RCAN2 in the serum of NAFLD mice and found a trend of increase but without a statistical difference (Supplementary Figure S3). This may be due to the insufficient number of mice.

### General Clinical Characteristics and Serum RCAN2 Concentration in the Study Population

In order to investigate the expression of serum RCAN2 in NAFLD patients, 346 non-NAFLD subjects and 369 NAFLD patients were enrolled in this study. The general characteristics of the study subjects are displayed in Table 1. The average age of all the subjects was 40.52 ± 10.58 years, including 464 males (64.90%) and 251 females (35.10%). Compared with the non-NAFLD group, the NAFLD group had a higher male/female ratio, BW, BMI, WC, HC, WHR, SBP, DBP, FBG, WBC, NEU, HGB, PLT, ALT, AST, TP, ALB, GGT, ALP, UA, CREA, TC, TG, LDL, HCY, and FLI (all *p* < 0.05) but lower AST/ALT, DBIL, eGFR, and HDL-C (all *p* < 0.05). No significant differences were found in age, GLO, A/G, TBIL, IBIL, and UREA (all *p* > 0.05).

**TABLE 1:** Clinical parameters of all participants in the study.

Measurements	Total	Non-NAFLD	NAFLD	*p*-value
Sample (n)	715	346	369	—
Male/Female	464/251	202/144	262/107	0.000
Age (years)	40.52 ± 10.58	40.14 ± 10.65	40.87 ± 10.51	0.321
BW (kg)	68.61 ± 12.84	61.94 ± 7.86	74.86 ± 13.45	0.000
BMI (kg/m2)	25.18 ± 3.62	23.06 ± 2.29	27.16 ± 3.51	0.000
WC (cm)	84.33 ± 9.49	79.12 ± 6.56	89.22 ± 9.21	0.000
HC (cm)	97.22 ± 6.78	94.40 ± 5.10	99.86 ± 7.09	0.000
WHR (cm/cm)	0.87 ± 0.06	0.84 ± 0.05	0.89 ± 0.05	0.000
SBP (mmHg)	122.08 ± 14.21	117.64 ± 13.16	126.24 ± 13.91	0.000
DBP (mmHg)	73.57 ± 10.10	70.65 ± 8.83	76.30 ± 10.45	0.000
FBG (mmol/L)	5.26 ± 1.23	4.94 ± 0.63	5.56 ± 1.54	0.000
WBC (*10^9/L)	6.37 ± 1.49	5.97 ± 1.37	6.74 ± 1.50	0.000
NEU (*10^9/L)	3.70 ± 1.13	3.49 ± 1.08	3.91 ± 1.14	0.000
HGB (g/L)	149.43 ± 14.44	145.51 ± 14.71	153.11 ± 13.18	0.000
PLT (*10^9/L)	234.352 ± 57.03	229.04 ± 54.77	239.34 ± 58.71	0.021
ALT (U/L)	30.64 ± 25.19	20.64 ± 8.77	40.01 ± 31.25	0.000
AST (U/L)	24.38 ± 10.70	21.35 ± 5.08	27.22 ± 13.46	0.000
AST/ALT	0.97 ± 0.39	1.15 ± 0.41	0.80 ± 0.27	0.000
TP (g/L)	72.33 ± 3.33	71.85 ± 3.37	72.78 ± 3.24	0.001
ALB (g/L)	46.48 ± 2.28	46.18 ± 2.32	46.75 ± 2.21	0.001
GLO (g/L)	25.86 ± 2.66	25.67 ± 2.50	26.04 ± 2.80	0.142
A/G	1.82 ± 0.21	1.82 ± 0.20	1.82 ± 0.22	0.927
TBIL (µmol/L)	14.73 ± 5.41	14.73 ± 4.64	14.73 ± 6.05	0.137
DBIL (µmol/L)	4.12 ± 1.54	4.23 ± 1.42	4.02 ± 1.65	0.007
IBIL (µmol/L)	10.61 ± 4.06	10.50 ± 3.41	10.72 ± 4.59	0.390
GGT (U/L)	35.87 ± 37.70	23.29 ± 20.22	47.67 ± 45.67	0.000
ALP (U/L)	73.27 ± 20.16	69.99 ± 19.80	76.35 ± 20.03	0.000
UREA (mol/L)	5.01 ± 1.09	4.94 ± 1.12	5.09 ± 1.05	0.099
UA (µmol/L)	351.95 ± 85.69	319.24 ± 75.71	382.62 ± 83.21	0.000
CREA (µmol/L)	66.70 ± 12.23	65.39 ± 12.43	67.93 ± 11.92	0.006
eGFR [ml/(min*1.73m^2^)]	122.58 ± 20.92	124.02 ± 20.74	121.22 ± 21.02	0.028
TC (mmol/L)	4.86 ± 0.90	4.71 ± 0.83	5.00 ± 0.93	0.000
TG (mmol/L)	1.80 ± 1.62	1.18 ± 0.59	2.38 ± 2.02	0.000
HDL-C (mmol/L)	1.30 ± 0.33	1.45 ± 0.35	1.16 ± 0.25	0.000
LDL-C (mmol/L)	3.22 ± 0.90	3.05 ± 0.83	3.37 ± 0.93	0.000
HCY (µmol/L)	12.26 ± 7.86	11.92 ± 7.44	12.58 ± 8.22	0.021
RCAN2 (ng/ml)	10.18 ± 4.56	8.79 ± 2.85	11.47 ± 5.42	0.000
NAFLD index FLI	36.27 ± 27.59	17.04 ± 13.75	54.31 ± 25.02	0.000

Continuous variables were expressed as mean ± standard deviation. Categorical variables were expressed as n/n. NAFLD, non-alcoholic fatty liver disease; BW, body weight; BMI, body mass index; WC, waist circumference; HC, hip circumference; WHR, waist to hip ratio; SBP, systolic blood pressure; DBP, diastolic blood pressure; FBG, fasting blood glucose; WBC, white blood cells; NEU, neutrophil; ALT, alanine aminotransferase; AST, aspartate aminotransferase; TP, total protein; ALB, albumin; GLO, globulin; A/G, Albumin-globulin-ratio; TBIL, total bilirubin; DBIL, direct bilirubin; IBIL, indirect bilirubin; GGT, gamma-glutamyl transpeptidase; ALP, alkaline phosphatase; UA, uric acid; CREA, creatinine; eGFR, estimated glomerular filtration rate; TC, total cholesterol; TG, triglyceride, HDL-C, high-density lipoprotein cholesterol; LDL-C, low-density lipoprotein cholesterol; HCY, homocysteine; RCAN2, a regulator of calcineurin 2; FLI, fatty liver index. *p < 0.05, **p < 0.01, ***p < 0.001 versus non-NAFLD group.

**FIGURE 2:**
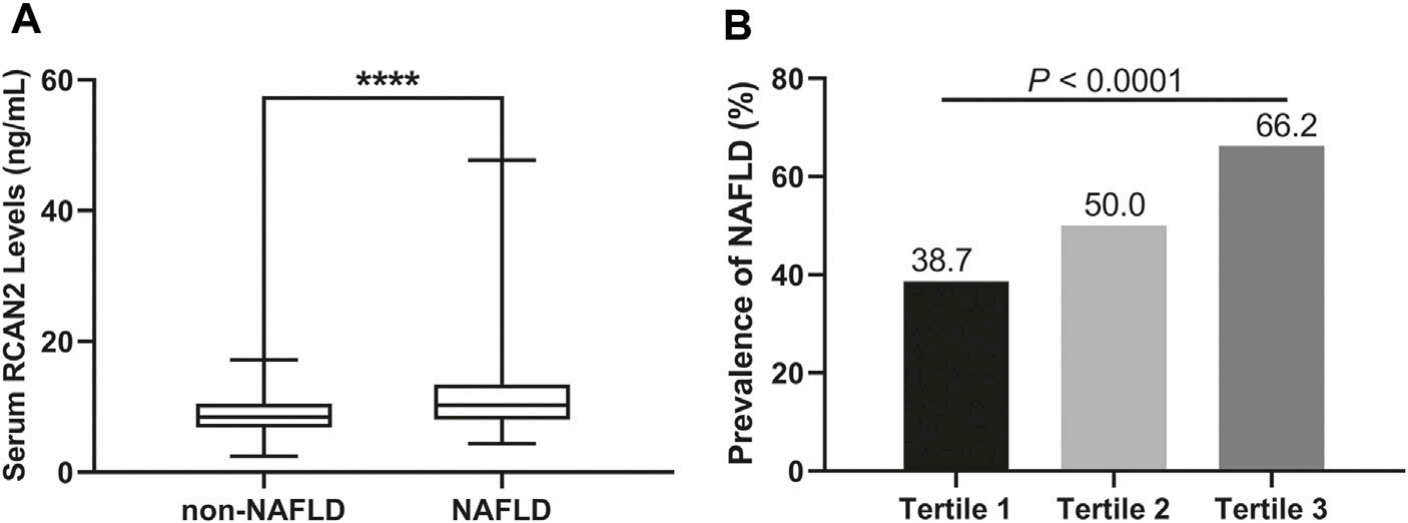
**(A)** Serum RCAN2 concentration in NAFLD patients and non-NAFLD subjects. **(B)** Prevalence of NAFLD by tertiles of serum RCAN2 levels in all participants. NAFLD, non-alcoholic fatty liver disease; RCAN2, a regulator of calcineurin 2. ****p < 0.0001 compared with non-NAFLD.

In all study participants, the distribution of serum RCAN2 levels was from 2.44–47.73 ng/mL. As shown in Table 1 and Figure 2A, the serum RCAN2 levels were significantly higher in the NAFLD group than those in the non-NAFLD group (11.47 ± 5.42 ng/ml vs. 8.79 ± 2.85 ng/ml, *p* = 0.000).

### Clinical Characteristics and Prevalence of NAFLD by Tertiles of Serum RCAN2 in all Study Subjects

In order to explore the changes of clinical parameters and prevalence of NAFLD under different RCAN2 concentration gradients in all study subjects, serum RCAN2 levels were divided into tertiles. As shown in Table 2 and Figure 2B, GGT, TC, TG, and the prevalence of NAFLD were gradually increased among the lowest tertile 1, median tertile 2, and the highest tertile 3 groups (all *p* < 0.05). The subjects in the highest tertile 3 had a higher male/female ratio, BW, BMI, WC, WHR, SBP, DBP, FBG, HGB, ALT, AST, TP, ALB, UA, LDL-C, and FLI than the lowest tertile 1 group, and a higher BW, BMI, SBP, and ALB than the median tertile 2 group (all *p* < 0.05).

**TABLE 2:** Clinical and biochemical characteristics by tertiles of serum RCAN2 level in all subjects.

Measurements	Tertile 1	Tertile 2	Tertile 3	*p*-value
Sample (n)	238	240	237	-
Male/Female	140/98	153/87	171/66**	0.009
Age (years)	39.31 ± 10.01	40.66 ± 10.89	41.59 ± 10.73	0.076
BW (kg)	67.69 ± 13.55	67.03 ± 10.91	71.13 ± 13.56**^##^	0.001
BMI (kg/m2)	24.85 ± 3.65	24.77 ± 3.11	25.92 ± 3.95**^#^	0.002
WC (cm)	83.29 ± 9.12	83.88 ± 9.40	85.83 ± 9.78*	0.015
HC (cm)	97.43 ± 6.50	96.56 ± 6.55	97.66 ± 7.25	0.240
WHR (cm/cm)	0.85 ± 0.06	0.87 ± 0.06	0.88 ± 0.05***	0.000
SBP (mmHg)	119.15 ± 13.09	121.80 ± 13.53	125.30 ± 15.29***^#^	0.000
DBP (mmHg)	71.35 ± 10.03	74.10 ± 9.46**	75.25 ± 10.43***	0.000
FBG (mmol/L)	5.12 ± 0.94	5.22 ± 1.00	5.45 ± 1.61*	0.014
WBC (*10^9/L)	6.28 ± 1.55	6.36 ± 1.46	6.46 ± 1.45	0.171
NEU (*10^9/L)	3.72 ± 1.15	3.70 ± 1.14	3.68 ± 1.12	0.935
HGB (g/L)	147.35 ± 15.11	149.05 ± 13.79	151.91 ± 14.09**	0.004
PLT (*10^9/L)	229.99 ± 56.80	235.24 ± 50.79	237.83 ± 62.89	0.524
ALT (U/L)	26.96 ± 20.02	29.70 ± 20.46	35.28 ± 32.51**	0.002
AST (U/L)	22.67 ± 7.32	23.87 ± 9.09	26.61 ± 14.17**	0.004
AST/ALT	1.03 ± 0.43	0.95 ± 0.37	0.93 ± 0.35*	0.015
TP (g/L)	71.77 ± 3.02	72.38 ± 3.36	72.84 ± 3.53**	0.006
ALB (g/L)	46.24 ± 2.16	46.33 ± 2.29	46.86 ± 2.35*^#^	0.011
GLO (g/L)	25.54 ± 2.46	26.05 ± 2.66	25.98 ± 2.84	0.143
A/G	1.83 ± 0.21	1.80 ± 0.21	1.82 ± 0.22	0.106
TBIL (µmol/L)	14.72 ± 5.57	14.60 ± 5.29	14.87 ± 5.39	0.842
DBIL (µmol/L)	4.30 ± 1.61	4.11 ± 1.47	3.95 ± 1.52*	0.032
IBIL (µmol/L)	10.43 ± 4.15	10.49 ± 3.98	10.93 ± 4.06	0.242
GGT (U/L)	30.36 ± 33.50	36.03 ± 41.89*	41.25 ± 36.56***^#^	0.000
ALP (U/L)	71.88 ± 20.02	73.32 ± 20.53	74.62 ± 19.91	0.299
UREA (mol/L)	4.89 ± 1.09	5.05 ± 1.14	5.09 ± 1.02	0.080
UA (µmol/L)	339.74 ± 81.43	347.82 ± 79.45	368.39 ± 93.40**	0.004
CREA (µmol/L)	65.67 ± 12.12	66.97 ± 12.33	67.47 ± 12.22	0.218
eGFR [ml/(min*1.73m2)]	123.77 ± 21.07	121.54 ± 20.37	122.42 ± 21.34	0.448
TC (mmol/L)	4.56 ± 0.80	4.86 ± 0.87***	5.17 ± 0.91***^###^	0.000
TG (mmol/L)	1.34 ± 0.80	1.58 ± 0.82**	2.49 ± 2.43***^###^	0.000
HDL-C (mmol/L)	1.34 ± 0.32	1.31 ± 0.35	1.26 ± 0.34*	0.020
LDL-C (mmol/L)	3.00 ± 0.79	3.28 ± 0.84**	3.38 ± 1.01***	0.000
HCY (µmol/L)	12.02 ± 7.80	12.14 ± 6.93	12.62 ± 8.77	0.450
RCAN2 (ng/ml)	6.45 ± 1.17	9.27 ± 0.80***	14.84 ± 4.93***^###^	0.000
NAFLD (%)	38.66	50.00*	66.24***^###^	0.000
NAFLD index FLI	28.70 ± 24.27	34.29 ± 25.98	45.88 ± 29.57***^###^	0.000

Continuous variables were expressed as mean ± standard deviation. Categorial variables were expressed as n (%). NAFLD, non-alcoholic fatty liver disease; BW, body weight; BMI, body mass index; WC, waist circumference; HC, hip circumference; WHR, waist to hip ratio; SBP, systolic blood pressure; DBP, diastolic blood pressure; FBG, fasting blood glucose; WBC, white blood cells; NEU, neutrophil; ALT, alanine aminotransferase; AST, aspartate aminotransferase; TP, total protein; ALB, albumin; GLO, globulin; A/G, Albumin-globulin-ratio; TBIL, total bilirubin; DBIL, direct bilirubin; IBIL, indirect bilirubin; GGT, gamma-glutamyl transpeptidase; ALP, alkaline phosphatase; UA, uric acid; CREA, creatinine; eGFR, estimated glomerular filtration rate; TC, total cholesterol; TG, triglyceride, HDL-C, high-density lipoprotein cholesterol; LDL-C, low-density lipoprotein cholesterol; HCY, homocysteine; RCAN2, regulator of calcineurin 2; FLI, fatty liver index. *p < 0.05, **p < 0.01, ***p < 0.001 versus tertile 1 group. ^#^ *p* < 0.05, ^##^ *p* < 0.01, ^###^ *p* < 0.001 versus tertile 2 group.

### Correlations and Regression of Serum RCAN2 Levels With Clinical Parameters in the Study Participants

In order to investigate the associations between serum RCAN2 levels and other clinical parameters, partial correlation analysis was performed. As displayed in Table 3, after adjustment for age and sex, serum RCAN2 levels were positively associated with BW (r = 0.157), BMI (r = 0.175), WC (r = 0.139), WHR (r = 0.167), SBP (r = 0.143), DBP (r = 0.120), FBG (r = 0.106), WBC (r = 0.096), ALT (r = 0.103), AST (r = 0.098), TP (r = 0.134), GLO (r = 0.123), GGT (r = 0.088), ALP (r = 0.080), UA (r = 0.224), TC (r = 0.263), TG (r = 0.475), LDL-C (r = 0.079), and FLI (r = 0.304) but negatively associated with AST/ALT (r = −0.097), A/G (r = −0.084), DBIL (r = −0.141), and HDL-C (r = −0.158) in all subjects (all *p* < 0.05). In the non-NAFLD group, serum RCAN2 was found to be positively associated with TP (r = 0.137), ALB (r = 0.129), TC (r = 0.191), TG (r = 0.182), and LDL-C (r = 0.119), and negatively associated with HC (r = −0.113) (all *p* < 0.05). In the NAFLD group, serum RCAN2 levels were positively associated with SBP (r = 0.105), GLO (r = 0.119), ALP (r = 0.127), UA (r = 0.208), TC (r = 0.266), TG (r = 0.458), and FLI (r = 0.198) but negatively associated with A/G (r = −0.110) and HDL-C (r = −0.108) (all *p* < 0.05).

**TABLE 3:** Partial correlation between serum RCAN2 levels and other parameters.

Measurements	Serum RCAN2 (age and sex-adjusted)					
	All subjects		Non-NAFLD		NAFLD	
—	r	*p*-value	r	*p*-value	r	*p*-value
BW (kg)	**0.157**	**0.000**	−0.030	0.577	0.029	0.584
BMI (kg/m2)	**0.175**	**0.000**	−0.046	0.400	0.046	0.384
WC (cm)	**0.139**	**0.000**	−0.057	0.289	0.005	0.931
HC (cm)	0.070	0.061	−**0.113**	**0.036**	−0.028	0.594
WHR (cm/cm)	**0.167**	**0.000**	0.031	0.570	0.049	0.351
SBP (mmHg)	**0.143**	**0.000**	0.010	0.859	**0.105**	**0.044**
DBP (mmHg)	**0.120**	**0.001**	0.051	0.350	0.054	0.305
FBG (mmol/L)	**0.106**	**0.005**	0.049	0.363	0.034	0.518
WBC (*10^9/L)	**0.096**	**0.010**	0.002	0.963	0.044	0.403
NEU (*10^9/L)	0.032	0.377	−0.092	0.089	0.014	0.785
HGB (g/L)	0.073	0.051	−0.006	0.919	0.010	0.848
PLT (*10^9/L)	0.072	0.053	0.105	0.052	0.017	0.741
ALT (U/L)	**0.103**	**0.006**	−0.022	0.686	−0.002	0.974
AST (U/L)	**0.098**	**0.009**	0.047	0.381	0.027	0.606
AST/ALT	**-0.097**	**0.010**	0.023	0.665	0.060	0.252
TP (g/L)	**0.134**	**0.000**	**0.137**	**0.011**	0.086	0.099
ALB (g/L)	0.054	0.148	**0.129**	**0.017**	−0.027	0.602
GLO (g/L)	**0.123**	**0.001**	0.074	0.173	**0.119**	**0.023**
A/G	−**0.084**	**0.026**	−0.017	0.759	−**0.110**	**0.035**
TBIL (µmol/L)	−0.027	0.467	0.003	0.955	−0.036	0.492
DBIL (µmol/L)	−**0.141**	**0.000**	−0.066	0.224	−**0.153**	**0.003**
IBIL (µmol/L)	0.016	0.663	0.031	0.568	0.006	0.907
GGT (U/L)	**0.088**	**0.019**	−0.035	0.517	0.006	0.912
ALP (U/L)	**0.080**	**0.032**	−0.104	0.055	**0.127**	**0.015**
UREA (mol/L)	0.037	0.324	−0.012	0.822	0.049	0.345
UA (µmol/L)	**0.224**	**0.000**	−0.019	0.730	**0.208**	**0.000**
CREA (µmol/L)	−0.009	0.804	−0.061	0.261	0.010	0.844
eGFR [ml/(min*1.73m2)]	0.017	0.647	0.080	0.140	−0.009	0.860
TC (mmol/L)	**0.263**	**0.000**	**0.191**	**0.000**	**0.266**	**0.000**
TG (mmol/L)	**0.475**	**0.000**	**0.182**	**0.001**	**0.458**	**0.000**
HDL-C (mmol/L)	−**0.158**	**0.000**	0.049	0.365	−**0.108**	**0.038**
LDL-C (mmol/L)	**0.079**	**0.034**	**0.119**	**0.027**	0.013	0.802
HCY (µmol/L)	−0.048	0.199	−0.023	0.667	−0.068	0.192
NAFLD index FLI	**0.304**	**0.000**	0.005	0.933	**0.198**	**0.000**

Partial correlation coefficients were used for age- and sex-adjusted data. Bold font indicated *p* < 0.05. RCAN2, regulator of calcineurin 2; NAFLD, non-alcoholic fatty liver disease; BW, body weight; BMI, body mass index; WC, waist circumference; HC, hip circumference; WHR, waist to hip ratio; SBP, systolic blood pressure; DBP, diastolic blood pressure; FBG, fasting blood glucose; WBC, white blood cells; NEU, neutrophil; ALT, alanine aminotransferase; AST, aspartate aminotransferase; TP, total protein; ALB, albumin; GLO, globulin; A/G, Albumin-globulin-ratio; TBIL, total bilirubin; DBIL, direct bilirubin; IBIL, indirect bilirubin; GGT, gamma-glutamyl transpeptidase; ALP, alkaline phosphatase; UA, uric acid; CREA, creatinine; eGFR, estimated glomerular filtration rate; TC, total cholesterol; TG, triglyceride, HDL-C, high-density lipoprotein cholesterol; LDL-C, low-density lipoprotein cholesterol; HCY, homocysteine; FLI, fatty liver index.

**FIGURE 3:**
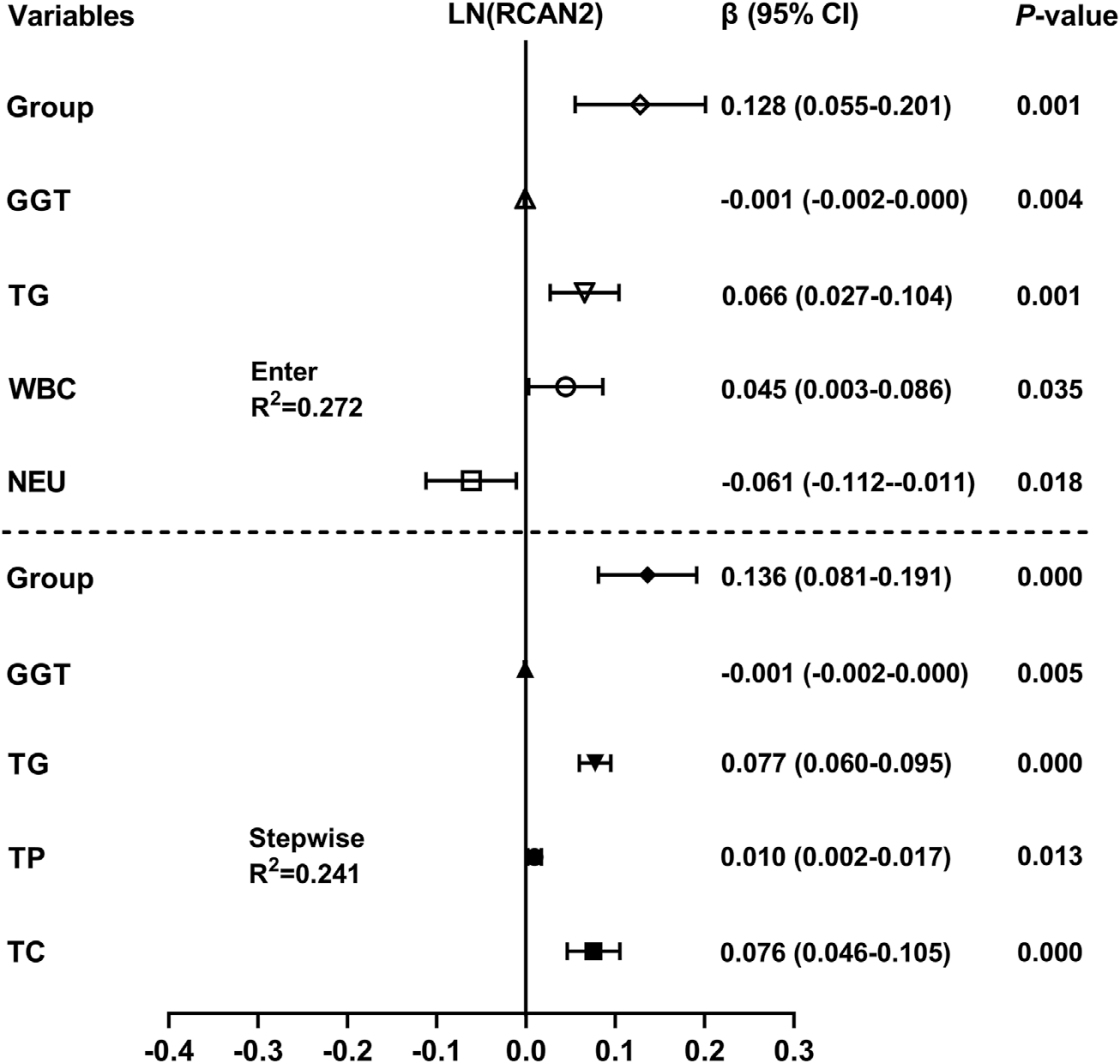
Multiple linear regression analysis of variables independently associated with serum RCAN2 levels in all participants. The regression coefficients (β) and 95% confidence interval (CI) were displayed. Serum RCAN2 levels were naturally logarithmic transformed before analysis. The group of statistic: 1 = non-NAFLD, 2 = NAFLD.

Liner regression analysis was performed to explore variables independently associated with serum RCAN2 levels. As shown in Figure 3, all factors enter and stepwise regression analyses showed that group, GGT, and TG were independent factors associated with serum RCAN2 levels.

### Association of Serum RCAN2 Levels and Risk of NAFLD

In order to further evaluate the association between serum RCAN2 levels and NAFLD risk, all subjects in the study were divided into three groups according to serum RCAN2 tertiles (lowest: < 8.03 ng/ml; median: 8.03–10.82 ng/ml; highest: ≥10.82 ng/ml). As displayed in Table 4, binary logistic regression analysis showed that subjects in the highest tertile of serum RCAN2 levels had a 2.114-fold higher risk of NAFLD than those in the lowest tertile levels (OR = 3.114, 95% CI = 2.141–4.531, *p* = 0.000). After adjusting for age, sex, BMI, SBP, DBP, FBG, WBC, NEU, ALT, AST, GGT, ALP, UA, and eGFR in the Model 2–4, the tendency still existed (OR = 2.912, 95% CI = 1.992–4.526, *p* = 0.000), (OR = 2.893, 95% CI = 1.715–4.881, *p* = 0.000), or (OR = 2.406, 95% CI = 1.324–4.373, *p* = 0.004). When further controlling for lipid profiles (TC, TG, LDL-C, and LDL-C) in Model 5, subjects in the highest tertile of serum RCAN2 levels also had a significantly increased risk of NAFLD (OR = 2.081, 95% CI = 1.084–3.995, *p* = 0.028). When considered as continuous variables, every 1-unit increase in serum RCAN2 levels was associated with 20.1, 19.3, 19.9, 18.9, and 17.5% increase, respectively, in risk of NAFLD prevalence in Model 1–5.

**TABLE 4:** Binary logistic regression of NAFLD risks according to tertiles or per 1-unit increases of serum RCAN2 concentrations.

	RCAN2 tertiles			Per 1-unit increase
	Lowest OR (95% CI)	Median OR (95% CI)	Highest OR (95% CI)	
Range (ng/ml)	<8.03	≥8.03–10.82	≥10.82	—
Model 1	1 (reference)	1.587 (1.103–2.282)	3.114 (2.141–4.531)	1.201 (1.145–1.260)
*p*-value	—	0.013	0.000	0.000
Model 2	1 (reference)	1.545 (1.071–2.229)	2.912 (1.992–4.526)	1.193 (1.136–1.252)
*p*-value	—	0.020	0.000	0.000
Model 3	1 (reference)	1.718 (1.045–2.825)	2.893 (1.715–4.881)	1.199 (1.121–1.282)
*p*-value	—	0.033	0.000	0.000
Model 4	1 (reference)	1.469 (0.842–2.562)	2.406 (1.324–4.373)	1.189 (1.102–1.283)
*p*-value	—	0.176	0.004	0.000
Model 5	1 (reference)	1.479 (0.825–2.651)	2.081 (1.084–3.995)	1.175 (1.083–1.275)
*p*-value	—	0.188	0.028	0.000

NAFLD, non-alcoholic fatty liver disease; RCAN2, regulator of calcineurin 2; OR, odds ratios; CI, confidence intervals. BMI, body mass index; WC, waist circumference; SBP, systolic blood pressure; DBP, diastolic blood pressure; FBG, fasting blood glucose; WBC, white blood cells; NEU, neutrophil; ALT, alanine aminotransferase; AST, aspartate aminotransferase; GGT, gamma-glutamyl transpeptidase; ALP, alkaline phosphatase; UA, uric acid; eGFR, estimated glomerular filtration rate; TC, total cholesterol; TG, triglyceride, HDL-C, high-density lipoprotein cholesterol; LDL-C, low-density lipoprotein cholesterol. Model 1 was not adjusted. Model 2: adjusted for age and sex. Model 3: adjusted for Model 2 + BMI, WC, SBP, DBP, FBG. Model 4: adjusted for Model 3 + WBC, NEU, ALT, AST, GGT, ALP, UA, eGFR. Model 5: adjusted for Model 4 + TC, TG, HDL-C, LDL-C.

### Diagnostic Value of Serum RCAN2 Levels and RCAN2/(AST/ALT) Ratio for NAFLD Risk

**FIGURE 4:**
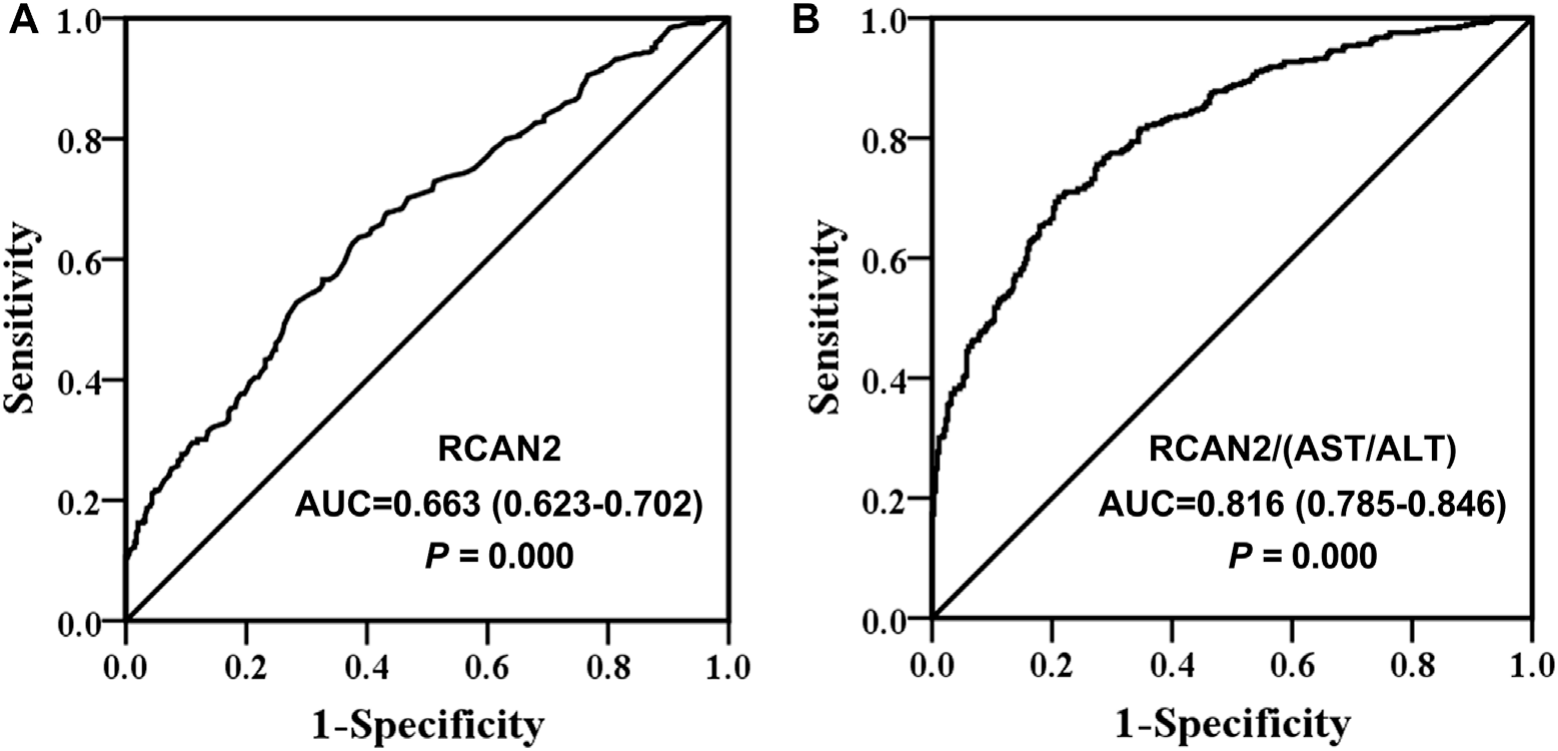
Receiver operating characteristic (ROC) curve analysis of serum RCAN2 levels **(A)** and serum RCAN2/(AST/ALT) ratio **(B)** in NAFLD patients and non-NAFLD subjects. NAFLD, non-alcoholic fatty liver disease; RCAN2, regulator of calcium 2; AUC, area under the curve.

Finally, ROC curve analysis was performed to investigate the predictive value of serum RCAN2 levels for NAFLD. The results revealed that best cutoff value for serum RCAN2 to predict NAFLD was 9.11 ng/ml (AUC = 0.663, 95% CI = 0.623–0.702, *p* = 0.000, sensitivity = 63.7%, specificity = 61.3%) (Figure 4A). In order to improve the diagnostic value, the factors (AST/ALT, A/G, DBIL, HDL-C) negatively correlated with serum RCAN2 levels in all study subjects were obtained by partial correlation analysis. Then, the ratios of serum RCAN2 levels to AST/ALT, A/G, DBIL, and HDL-C were calculated. ROC curve analysis was performed again to explore the predictive value of these ratios for NAFLD. As displayed in Figure 4B, the serum RCAN2/(AST/ALT) ratio showed improved predictive accuracy (AUC = 0.816, 95% CI = 0.785–0.846, *p* = 0.000, sensitivity = 70.2%, specificity = 78.9%).

## Discussion

This study was the first to investigate the association between serum RCAN2 levels and NAFLD in humans. The main innovation of the current study is that we first demonstrated that RCAN2 was highly expressed in the liver of mice with fatty liver by qRT-PCR and Western blot, and subsequently confirmed that serum RACN2 levels were also significantly elevated in NAFLD patients. Meanwhile, the results of the present study showed that serum RCAN2 levels were positively correlated with the prevalence of NAFLD. Systemic knockout of RCAN2 (RCAN2^−/−^) has been reported to prevent hepatic steatosis in mice fed a high-fat diet (Sun et al., 2011; Zhao et al., 2016). Recently, RCAN2 was found to be a target gene of peroxisome proliferator-activated receptor gamma (PPARγ) (Yuan et al., 2021), which regulates hepatic lipid deposition in mice with diet-induced obesity (Chen et al., 2022). Taken together, RCAN2 may be a pathogenic driver of NAFLD. It has been reported that the global knockout of RCAN2 can significantly reduce the liver weight in obese mice induced by a high-fat diet by reducing the food intake. This is probably the main reason that RCAN2−/−mice did not develop typical features of fatty liver on a high-fat diet (Sun et al., 2011; Zhao et al., 2016). Both RCAN2-3 and RCAN2-1 were knocked out in these studies, and it was mainly the downregulation of RCAN2-3 rather than RCAN2-1 that caused the reduction of food intake. RCAN2-3 was expressed only in the brain, while RCAN2-1 was widely expressed in the heart, muscle, kidney, and liver (Cao et al., 2002; Mizuno et al., 2004). Systemic knockout of RCAN2-3 and RCAN2-1 in the whole organism may obscure the role of RCAN2-1 in specific tissues. In the current study, we found that RCAN2-1 was significantly upregulated in the liver tissues of the genetic and HFD-induced steatosis mouse model. Whether liver specific-knockout of RCAN2-1 is involved in the development of NAFLD deserves further study.

RCAN2 was first recognized as a thyroid hormone responsive gene (Miyazaki et al., 1996). In subsequent studies, only RCAN2-3 was regulated by T3 in a human skin fibroblast cell line (Cao et al., 2002). Hypothyroidism significantly decreased the mRNA expression of RCAN2-3 in the brain and had no effect on RCAN2-1, while hyperthyroidism had no influence on RCAN2-1 and RCAN2-3. In the heart, hyperthyroidism upregulated the expression of RCAN2-1, whereas hypothyroidism has no effect on RCAN2-1. However, in the liver, exogenous T3 had no effect on the expression of RCAN2-1 (Mizuno et al., 2004). In animal or cell experiments, T3 was found to reduce hepatocyte lipid storage by promoting fibroblast growth factor 21 (Adams et al., 2010). In the present study, T3 was within the normal range in all the subjects tested for T3 (data not shown). Moreover, partial correlation analysis (age and sex-adjusted) (r = 0.042, *p* = 0.603) and multiple linear regression analysis= 0.768, 95% CI = −2.261–3.796, *p* = 0.617) also showed that serum RCAN2 levels were not correlated with T3. Therefore, these results suggested that elevated serum RCAN2 levels in NAFLD patients may not be associated with T3.

In all study subjects, partial correlation analysis showed that serum RCAN2 levels were positively correlated with BW, BMI, WC, WHR, SBP, DBP, FBG, WC, ALT, AST, TP, GLO, GGT, ALP, UA, TC, TG, and LDL-C, and negatively associated with AST/ALT ratio, A/G ratio, DBIL, and HDL-C. In non-NAFLD participants, the partial correlation analysis showed that serum RCAN2 levels were positively associated with TP, ALB, TC, TG, and LDL-C and negatively associated with HC. In NAFLD patients, after age and sex adjustment, serum RCAN2 was still positively correlated with SBP, GLO, ALP, UA, TC, and TG. Based on these results, we found that RCAN2 was significantly positively correlated with TC and TG in all participants, non-NAFLD subjects, and NAFLD patients, and had a closer correlation with TG, especially in all participants (r = 0.475, *p* = 0.000) and NAFLD patients (r = 0.458, *p* = 0.000). Additionally, the close relationship between serum RCAN2 and TG was further confirmed by multiple linear regression analysis. It is well known that NAFLD is based on an abnormal increase in the hepatic lipid content (mainly TG), followed by pathological processes such as hepatocellular injury, inflammation, or fibrosis (Donnelly et al., 2005; Friedman et al., 2018). From these findings, we hypothesized that RCAN2 promotes the occurrence of NAFLD by affecting the storage of TG in the liver, but more studies are needed to verify this relationship and clarify the specific mechanism. Given that NASH is a more serious process with inflammation, hepatocyte damage, or fibrosis, it has also been reported that upregulation of RCAN2 promotes inflammation and apoptosis (Luo et al., 2022). Therefore, future studies are necessary to explore the relationship between RCAN2 and fibrosis and inflammatory factors such as C-reactive protein.

Additionally, binary logistic regression analysis showed that subjects in the highest tertile of serum RCAN2 levels had a 2.114-fold higher risk of NAFLD than those in the lowest RCAN2 tertile. The increased risk of NAFLD was also observed at the highest RCAN2 levels after adjusting for age and sex in Model 2, and after further controlling BMI, WC, BP, FBG in Model 3, as well as adjusting for WBC, NEU, ALT, AST, GGT, ALP, UA, and eGFR in Model 4, and even adjusting for TC, TG, HDL-C, and LDL-C in Model 5. Similarly, per 1-unit increase in serum RCAN2 levels was also positively associated with the risk of NAFLD prevalence in Models 1–5. These results increase the possibility that elevated serum RCAN2 levels may serve as a novel biomarker for NAFLD. Further analyses using ROC curves found that serum RCAN2 may be a candidate biomarker for the diagnosis of NAFLD (AUC 0.663, sensitivity 63.7%, and specificity 61.3%). However, the diagnostic role of RCAN2 for NAFLD still needs to be verified in large-scale prospective studies in the future, especially in other ethnic groups and in NAFLD patients diagnosed by liver biopsy. In this study, we also found that serum RCAN2/(AST/ALT) ratio had a better diagnostic accuracy than serum RCAN2 levels, with an AUC of 0.816 and 70.2% sensitivity and 78.9% specificity.

In the present study, there are still some limitations. First, prospective studies are needed to illustrate the causal relationship between the serum RCAN2 levels and NAFLD. In addition, drug-intervention studies may also be used to explore the therapeutic effect of activation or inhibition of RACN2 on fatty liver in humans or animals. However, there are few studies on RCAN2 at present, we have to start with animal or cell research and explore the role and mechanism of RCAN2 in hepatocyte steatosis model through RNAi, overexpression plasmid, adeno-associated virus, and liver specific knockout. Second, hepatic steatosis was diagnosed by abdominal ultrasound rather than liver biopsy, which can lead to missed or misdiagnosis and cannot determine the severity of NAFLD. Third, animal studies have shown that RCAN2 can increase the food intake. In this clinical study, diet-related information, such as the type and frequency of eating, was not collected. Therefore, the relationship between RCAN2 and food intake was not established. Fourth, the association between serum RCAN2 levels and the progression of the disease cannot be demonstrated due to the lack of data on the diagnosis of NASH/fibrosis. Finally, all subjects were recruited from a physical examination center, which may lead to a selection bias.

In conclusion, the present study found that the serum RCAN2 levels were increased in NAFLD patients and were positively associated with the risk of NAFLD. Serum RCAN2, especially the serum RCAN2/(AST/ALT) ratio might be a novel diagnostic biomarker for NAFLD. In the future, prospective longitudinal studies and basic research, especially tissue-specific knockout studies of RCAN2, are needed to confirm the role of RCAN2 in the development of NAFLD.

## Data Availability

The original contributions presented in the study are included in the article/Supplementary Material, further inquiries can be directed to the corresponding authors.

## Glossary

A/G: Albumin-globulin-ratio
ALB: Albumin
ALP: Alkaline phosphatase
ALT: Alanine aminotransferase
AST: Aspartate aminotransferase
AUC: Area under the curve
BMI: Body mass index
Crea: Creatinine
DBP: Diastolic blood pressure
FBG: Fasting blood glucose
FLI: Fatty liver index
GGT: Gamma-glutamyl transpeptidase
GLO: Globulin
HC: Hip circumference
HCY: Homocysteine
HDL-C: High-density lipoprotein cholesterol
LDL-C: Low-density lipoprotein cholesterol
NAFLD: Non-alcoholic fatty liver disease
NASH: Non-alcoholic steatohepatitis
NEU: Neutrophil
qRT-PCR: Quantitative real-time PCR
SBP: Systolic blood pressure
TC: Total cholesterol
TG: Triglyceride
TP: Total protein
UA: Uric acid
WBC: White blood cells
WC: Waist circumference
WHR: Waist to hip ratio
RCAN2: Regulator of calcineurin 2

## References

[B1] Adams A. C. Astapova I. Fisher F. M. Badman M. K. Kurgansky K. E. Flier J. S. (2010). Thyroid Hormone Regulates Hepatic Expression of Fibroblast Growth Factor 21 in a PPARalpha-dependent Manner. J. Biol. Chem. 285 (19), 14078–14082. 10.1074/jbc.C110.107375 20236931 PMC2863226

[B2] Cao X. Kambe F. Miyazaki T. Sarkar D. Ohmori S. Seo H. (2002). Novel Human ZAKI-4 Isoforms: Hormonal and Tissue-specific Regulation and Function as Calcineurin Inhibitors. Biochem. J. 367 (Pt 2), 459–466. 10.1042/BJ20011797 12102656 PMC1222895

[B3] Chalasani N. Younossi Z. Lavine J. E. Diehl A. M. Brunt E. M. Cusi K. (2012). The Diagnosis and Management of Non-alcoholic Fatty Liver Disease: Practice Guideline by the American Association for the Study of Liver Diseases, American College of Gastroenterology, and the American Gastroenterological Association. Hepatology 55 (6), 2005–2023. 10.1002/hep.25762 22488764

[B4] Chalasani N. Younossi Z. Lavine J. E. Charlton M. Cusi K. Rinella M. (2018). The Diagnosis and Management of Nonalcoholic Fatty Liver Disease: Practice Guidance from the American Association for the Study of Liver Diseases. Hepatology 67 (1), 328–357. 10.1002/hep.29367 28714183

[B5] Chen Z. Y. Sun Y. T. Wang Z. M. Hong J. Xu M. Zhang F. T. (2022). Rab2A Regulates the Progression of Nonalcoholic Fatty Liver Disease Downstream of AMPK-TBC1D1 axis by Stabilizing PPARγ. Plos Biol. 20 (1), e3001522. 10.1371/journal.pbio.3001522 35061665 PMC8809606

[B6] Davies K. J. Ermak G. Rothermel B. A. Pritchard M. Heitman J. Ahnn J. (2007). Renaming the DSCR1/Adapt78 Gene Family as RCAN: Regulators of Calcineurin. FASEB J. 21 (12), 3023–3028. 10.1096/fj.06-7246com 17595344

[B7] Donnelly K. L. Smith C. I. Schwarzenberg S. J. Jessurun J. Boldt M. D. Parks E. J. (2005). Sources of Fatty Acids Stored in Liver and Secreted via Lipoproteins in Patients with Nonalcoholic Fatty Liver Disease. J. Clin. Invest. 115 (5), 1343–1351. 10.1172/JCI23621 15864352 PMC1087172

[B8] Fan J. G. Jia J. D. Li Y. M. Wang B. Y. Lu L. G. Shi J. P. (2011). Guidelines for the Diagnosis and Management of Nonalcoholic Fatty Liver Disease: Update 2010: (Published in Chinese on Chinese Journal of Hepatology 2010; 18:163-166). J. Dig. Dis. 12 (1), 38–44. 10.1111/j.1751-2980.2010.00476.x 21276207

[B9] Friedman S. L. Neuschwander-Tetri B. A. Rinella M. Sanyal A. J. (2018). Mechanisms of NAFLD Development and Therapeutic Strategies. Nat. Med. 24 (7), 908–922. 10.1038/s41591-018-0104-9 29967350 PMC6553468

[B10] Fuentes J. J. Genescà L. Kingsbury T. J. Cunningham K. W. Pérez-Riba M. Estivill X. (2000). DSCR1, Overexpressed in Down Syndrome, Is an Inhibitor of Calcineurin-Mediated Signaling Pathways. Hum. Mol. Genet. 9 (11), 1681–1690. 10.1093/hmg/9.11.1681 10861295

[B11] Gao P. Zhang H. Zhang Q. Fang X. Wu H. Wang M. (2019). Caloric Restriction Exacerbates Angiotensin II-Induced Abdominal Aortic Aneurysm in the Absence of P53. Hypertension 73 (3), 547–560. 10.1161/HYPERTENSIONAHA.118.12086 30686087

[B12] Gollogly L. K. Ryeom S. W. Yoon S. S. (2007). Down Syndrome Candidate Region 1-like 1 (DSCR1-L1) Mimics the Inhibitory Effects of DSCR1 on Calcineurin Signaling in Endothelial Cells and Inhibits Angiogenesis. J. Surg. Res. 142 (1), 129–136. 10.1016/j.jss.2006.10.011 17610901 PMC1995402

[B13] Hattori Y. Sentani K. Shinmei S. Oo H. Z. Hattori T. Imai T. (2019). Clinicopathological Significance of RCAN2 Production in Gastric Carcinoma. Histopathology 74 (3), 430–442. 10.1111/his.13764 30307052

[B14] Kingsbury T. J. Cunningham K. W. (2000). A Conserved Family of Calcineurin Regulators. Genes Dev. 14 (13), 1595–1604. 10.1101/gad.14.13.1595 10887154 PMC316734

[B15] Luo Y. Y. Xu H. T. Yang Z. Q. Lin X. F. Zhao F. L. Huang Y. S. (2022). Long Non-coding RNA MALAT1 Silencing Elevates microRNA-26a-5p to Ameliorate Myocardial Injury in Sepsis by Reducing Regulator of Calcineurin 2. Arch. Biochem. Biophys. 15, 715. 10.1016/j.abb.2021.109047 34619102

[B16] Mammarella E. Zampieri C. Panatta E. Melino G. Amelio I. (2021). NUAK2 and RCan2 Participate in the P53 Mutant Pro-tumorigenic Network. Biol. Direct 16 (1), 11. 10.1186/s13062-021-00296-5 34348766 PMC8335924

[B17] Miyazaki T. Kanou Y. Murata Y. Ohmori S. Niwa T. Maeda K. (1996). Molecular Cloning of a Novel Thyroid Hormone-Responsive Gene, ZAKI-4, in Human Skin Fibroblasts. J. Biol. Chem. 271 (24), 14567–14571. 10.1074/jbc.271.24.14567 8662924

[B18] Mizuno Y. Kanou Y. Rogatcheva M. Imai T. Refetoff S. Seo H. (2004). Genomic Organization of Mouse ZAKI-4 Gene that Encodes ZAKI-4 Alpha and Beta Isoforms, Endogenous Calcineurin Inhibitors, and Changes in the Expression of These Isoforms by Thyroid Hormone in Adult Mouse Brain and Heart. Eur. J. Endocrinol. 150 (3), 371–380. 10.1530/eje.0.1500371 15012624

[B19] Negro F. (2020). Natural History of NASH and HCC. Liver Int. 40 (Suppl. 1), 72–76. 10.1111/liv.14362 32077608

[B20] Niitsu H. Hinoi T. Kawaguchi Y. Sentani K. Yuge R. Kitadai Y. (2016). KRAS Mutation Leads to Decreased Expression of Regulator of Calcineurin 2, Resulting in Tumor Proliferation in Colorectal Cancer. Oncogenesis 5 (8), e253. 10.1038/oncsis.2016.47 27526107 PMC5007825

[B21] Parthasarathy G. Revelo X. Malhi H. (2020). Pathogenesis of Nonalcoholic Steatohepatitis: An Overview. Hepatol. Commun. 4 (4), 478–492. 10.1002/hep4.1479 32258944 PMC7109346

[B22] Rothermel B. Vega R. B. Yang J. Wu H. Bassel-Duby R. Williams R. S. (2000). A Protein Encoded within the Down Syndrome Critical Region Is Enriched in Striated Muscles and Inhibits Calcineurin Signaling. J. Biol. Chem. 275 (12), 8719–8725. 10.1074/jbc.275.12.8719 10722714

[B23] Siddiq A. Miyazaki T. Takagishi Y. Kanou Y. Hayasaka S. Inouye M. (2001). Expression of ZAKI-4 Messenger Ribonucleic Acid in the Brain during Rat Development and the Effect of Hypothyroidism. Endocrinology 142 (5), 1752–1759. 10.1210/endo.142.5.8156 11316738

[B24] Strippoli P. Petrini M. Lenzi L. Carinci P. Zannotti M. (2000). The Murine DSCR1-like (Down Syndrome Candidate Region 1) Gene Family: Conserved Synteny with the Human Orthologous Genes. Gene 257 (2), 223–232. 10.1016/s0378-1119(00)00407-8 11080588

[B25] Sun X. Y. Hayashi Y. Xu S. Kanou Y. Takagishi Y. Tang Y. P. (2011). Inactivation of the Rcan2 Gene in Mice Ameliorates the Age- and Diet-Induced Obesity by Causing a Reduction in Food Intake. PLoS One 6 (1), e14605. 10.1371/journal.pone.0014605 21298050 PMC3029291

[B26] Tan L. J. Jung H. Kim S. A. Shin S. (2021). The Association between Coffee Consumption and Nonalcoholic Fatty Liver Disease in the South Korean General Population. Mol. Nutr. Food Res. 65 (18), e2100356. 10.1002/mnfr.202100356 34319647

[B27] Younossi Z. M. Koenig A. B. Abdelatif D. Fazel Y. Henry L. Wymer M. (2016). Global Epidemiology of Nonalcoholic Fatty Liver Disease-Meta-Analytic Assessment of Prevalence, Incidence, and Outcomes. Hepatology 64 (1), 73–84. 10.1002/hep.28431 26707365

[B28] Yuan H. Suzuki S. Hirata-Tsuchiya S. Sato A. Nemoto E. Saito M. (2021). PPARγ-Induced Global H3K27 Acetylation Maintains Osteo/Cementogenic Abilities of Periodontal Ligament Fibroblasts. Int. J. Mol. Sci. 22 (16), 8646. 10.3390/ijms22168646 34445348 PMC8395443

[B29] Zhao J. Li S. W. Gong Q. Q. Ding L. C. Jin Y. C. Zhang J. (2016). A Disputed Evidence on Obesity: Comparison of the Effects of Rcan2(-/-) and Rps6kb1(-/-) Mutations on Growth and Body Weight in C57BL/6J Mice. J. Zhejiang Univ. Sci. B 17 (9), 657–671. 10.1631/jzus.B1600276 27604858 PMC5018613

