# Supplementary material for: Elevated Serum Regulator of Calcineurin 2 is Associated With an Increased Risk of Non-Alcoholic Fatty Liver Disease
Source: Front Pharmacol. 2022 Mar 16;13:840764. doi: 10.3389/fphar.2022.840764 (PMC8967172; doi:10.3389/fphar.2022.840764)
Supplement: Supplementary file 1 [file DataSheet2.PDF]

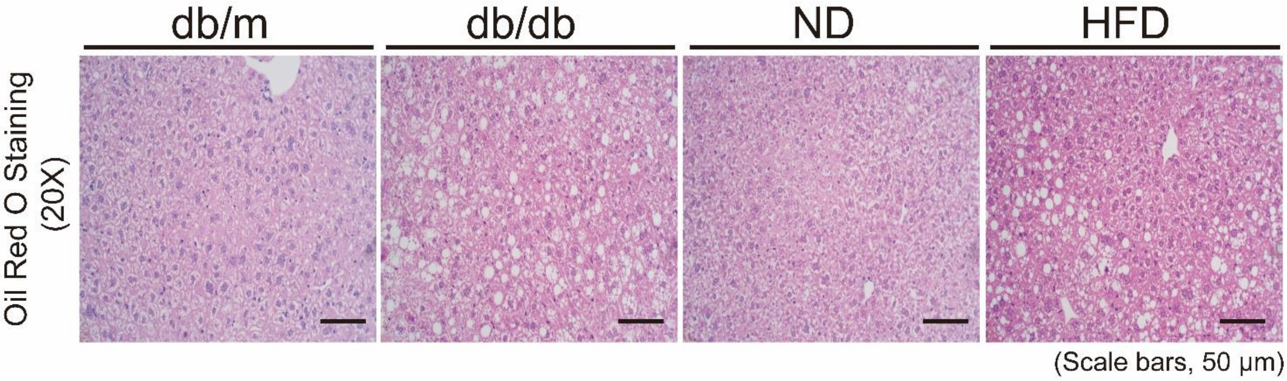

Fig S1. Representative gross morphology of the mouse livers, H&E staining of liver sections.

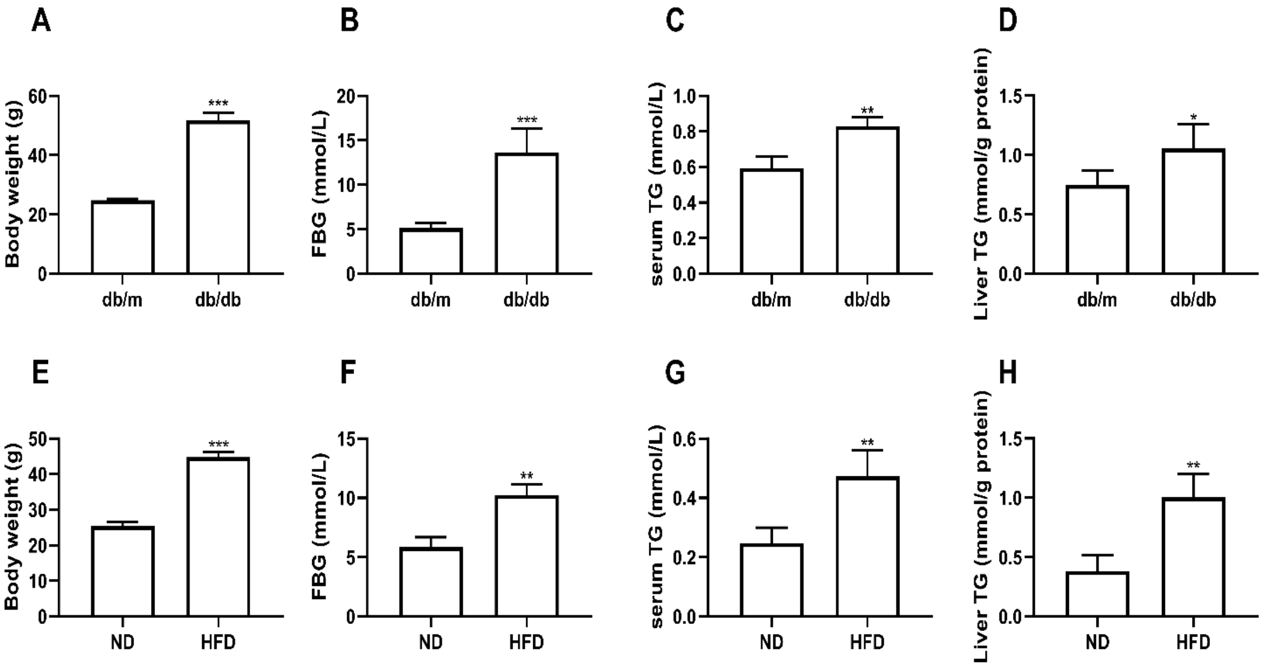

Fig S2. The metabolic parameters of NAFLD mouse model. (A) body weight, (B) fasting blood glucose, (C) serum TG levels, (D) liver TG levels in db/db mice vs db/m mice; (E) body weight, (F) fasting blood glucose, (G) serum TG levels, (H) liver TG levels in HFD mice vs ND mice. ND, normal diet; HFD, high fat diet. The data was presented as mean ± SD. *$P < 0.05$, **$P < 0.01$, ***$P < 0.001$ vs db/m or ND.

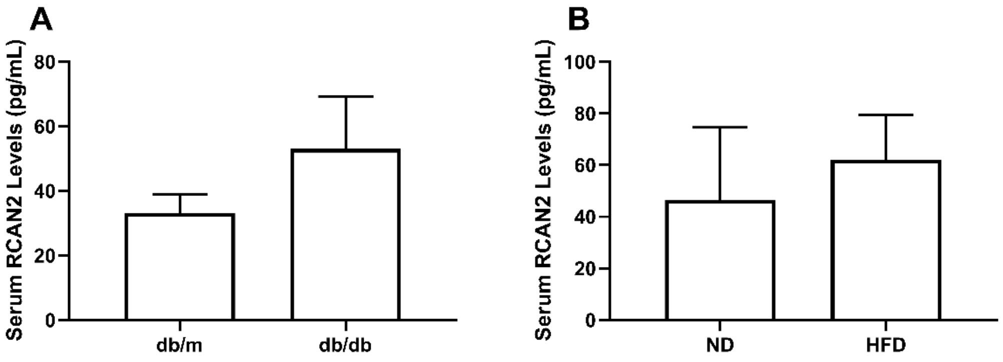

Fig S3. The concentration of serum RCAN2 in db/db mice vs db/m mice (A) and in HFD mice vs ND mice (B). ND, normal diet; HFD, high fat diet.
