# Supplementary material for: Elevated Serum Regulator of Calcineurin 2 is Associated With an Increased Risk of Non-Alcoholic Fatty Liver Disease
Source: Front Pharmacol. 2022 Mar 16;13:840764. doi: 10.3389/fphar.2022.840764 (PMC8967172; doi:10.3389/fphar.2022.840764)
Supplement: Supplementary file 2 [file DataSheet1.docx]

**Table S1. Quantitative real-time polymerase chain reaction primers.**

| Gene | Species | Forward primer (5'-3') | Reverse primer (5'-3') |
| --- | --- | --- | --- |
| RCAN2 | Mouse | ACCTATGATGAATGTGTGACGT | TTAGCTTCTTCCCTCTGAACTG |
| RCAN2 | Human | CTGCCAAACAGTTTCTCATCTC | CACAGCATAGAGGAGGTCATAG |
| GAPDH | Mouse | ACTCCACTCACGGCAAATTC | TCTCCATGGTGGTGAAGACA |
| GAPDH | Human | TCGCTCTCTGCTCCTCCTGTTC | CCGTTGACTCCGACCTTCACCT |

**Table S2. The characteristics of 15 DEGs using RNA sequencing for the livers of db/db and db/m mice**

| Gene | Gene annotation | db/db-FPKM | db/m-FPKM | log2(FC) | *P*-adjusted |
| --- | --- | --- | --- | --- | --- |
| MUP7 | Major urinary protein 7 | 3.565 | 8550.058 | -11.195 | 2.93E-90 |
| HSD3B5 | Hydroxy-delta-5-steroid dehydrogenase, 3 beta- and steroid delta-isomerase 5 | 0.034 | 15.788 | -8.822 | 8.71E-62 |
| MUP12 | Major urinary protein 12 | 45.119 | 4686.442 | -6.709 | 5.50E-57 |
| HSPA1B | Heat shock protein family a (hsp70) member 1b | 1.264 | 50.852 | -5.331 | 2.19E-23 |
| SERPINE2 | Serpin family e member 2 | 0.317 | 9.601 | -4.937 | 1.38E-24 |
| SLC25A30 | Solute carrier family 25 member 30 | 4.924 | 82.049 | -4.051 | 5.23E-23 |
| SDR9C7 | Short chain dehydrogenase/reductase family 9c member 7 | 1.340 | 21.406 | -3.944 | 5.58E-27 |
| SUSD4 | Sushi domain containing 4 | 1.122 | 10.515 | -3.283 | 2.57E-26 |
| CRELD2 | Cysteine rich with egf like domains 2 | 5.984 | 49.943 | -3.073 | 1.59E-28 |
| C8A | Complement c8 alpha chain | 26.491 | 190.371 | -2.862 | 5.56E-23 |
| GSTT3 | Glutathione s-transferase, theta 3 | 159.751 | 18.808 | 2.955 | 1.61E-30 |
| RAET1D | Retinoic acid early transcript delta | 66.419 | 6.777 | 3.281 | 6.40E-33 |
| RCAN2 | Regulator of calcineurin 2 | 21.531 | 1.007 | 4.393 | 6.21E-27 |
| SMPD3 | Sphingomyelin phosphodiesterase 3 | 7.252 | 0.126 | 5.451 | 4.55E-54 |
| FAM83F | Family with sequence similarity 83 member f | 2.630 | 0.014 | 7.497 | 6.79E-50 |

FC, fold change.

**Table S3. Expression trend of 15 DEGs in RNA sequencing analysis and qRT-PCR validation.**

| Gene | db/db vs. db/m  RNA-seq | db/db vs. db/m  RT-qPCR | HFD vs. ND  RT-qPCR | PA vs. BSA  RT-qPCR |
| --- | --- | --- | --- | --- |
| MUP7 | Down | Down | NS | - |
| HSD3B5 | Down | Down | Up | - |
| MUP12 | Down | Down | NS | - |
| HSPA1B | Down | Down | NS | - |
| SERPINE2 | Down | Down | NS | - |
| SLC25A30 | Down | Down | NS | - |
| SUSD4 | Down | NS | NS | - |
| CRELD2 | Down | Down | Up | - |
| SDR9C7 | Down | Down | NS | - |
| C8A | Down | Down | Up | - |
| GSTT3 | Up | Up | NS | - |
| RAET1D | Up | NS | NS | - |
| **RCAN2** | **Up** | **Up** | **Up** | **Up** |
| SMPD3 | Up | Up | NS | - |
| FAM83F | Up | NS | NS | **-** |

DEGs, differentially expressed genes; qRT-PCR, quantitative real-time PCR; ND, normal diet; HFD, high fat diet; BSA, bovine serum albumin; PA, palmitic acid.
